# The Value of Baseline [^18^F]FDG-PET in Predicting the Progression-Free Survival in Patients with Thymic Epithelial Tumours: A Systematic Review and Meta-Analysis

**DOI:** 10.3390/diagnostics15192458

**Published:** 2025-09-26

**Authors:** Alberto Miceli, Maria Librando, Francesco Dondi, Lorenzo Jonghi-Lavarini, Adriana D’Antonio, Antonio Mura, Anna Giulia Nappi, Guido Rovera, Maria Silvia De Feo, Giulia Santo, Francesco Lanfranchi

**Affiliations:** 1 Nuclear Medicine Unit, Azienda Ospedaliero-Universitaria SS. Antonio e Biagio e Cesare Arrigo, 15121 Alessandria, Italy; 2 Nuclear Medicine Unit, Department of Biomedical and Dental Sciences and Morpho-Functional Imaging, University of Messina, 98122 Messina, Italy; marialibrando@hotmail.it; 3 Division of Nuclear Medicine, Università degli Studi di Brescia and ASST Spedali Civili di Brescia, 25123 Brescia, Italy; francesco.dondi@unibs.it; 4 Department of Radiotherapy and Nuclear Medicine, ASST-Cremona, 26100 Cremona, Italy; lorenzomaria.jonghi-lavarini@asst-cremona.it; 5 Department of Advanced Biomedical Sciences, University Federico II, 80131 Naples, Italy; a.dantonio62@gmail.com; 6 Unit of Nuclear Medicine, Department of Medicine, Surgery and Pharmacy, University of Sassari, 07100 Sassari, Italy; a.mura203@studenti.uniss.it; 7 Nuclear Medicine Unit, Interdisciplinary Department of Medicine, University of Bari “Aldo Moro”, Piazza Giulio Cesare 11, 70124 Bari, Italy; anna.giulia.nappi@gmail.com; 8 Department of Medical Sciences, University of Turin, 10126 Turin, Italy; guido.rovera@unito.it; 9 Department of Radiological Sciences, Oncology and Anatomo Pathology, Sapienza University of Rome, 00151 Rome, Italy; mariasilvia.defeo@uniroma1.it; 10 Department of Experimental and Clinical Medicine, “Magna Graecia” University of Catanzaro, 88100 Catanzaro, Italy; giulia.santo@unicz.it; 11 Department of Experimental Medicine (DIMES), University of Genoa, 16132 Genoa, Italy; dr.francescolanfranchi@gmail.com

**Keywords:** thymic epithelial tumours, thymoma, thymic carcinoma, positron emission tomography, prognosis, progression-free survival

## Abstract

**Background/Objectives:** [^18^F]FDG-PET is often used for staging thymic epithelial tumours (TETs). However, its prognostic role remains uncertain. The aim of this present systematic review and meta-analysis is to assess the prognostic value of baseline [^18^F]FDG-PET-derived semiquantitative parameters in predicting progression-free survival (PFS) in patients with TETs. **Methods:** A systematic review and meta-analysis were conducted according to PRISMA guidelines. PubMed, Embase, and Scopus databases were searched up to 30 May 2025. Studies evaluating the prognostic impact of [^18^F]FDG-PET parameters on PFS in TETs were included. Pooled hazard ratios (HRs) with 95% confidence intervals (CIs) were calculated. **Results:** Six retrospective studies involving 593 patients were included. Maximum standardized uptake value (SUVmax), analysed as a continuous variable in four studies, significantly predicted worse PFS (HR: 1.18, 95% CI: 1.08–1.29, *p* < 0.001), with high inter-study heterogeneity (I^2^ = 79.7%). When dichotomized (two studies), higher SUVmax was associated with significantly poorer PFS (HR: 9.00, 95% CI: 2.93–27.71). Similarly, mean SUV (SUVmean) as a continuous predictor was also significantly associated with impaired PFS (HR: 1.41, 95% CI: 1.25–1.59), but only two studies assessed this parameter. Conversely, metabolic tumour volume (MTV) and total lesion glycolysis (TLG), both assessed as continuous prognosticators, did not show a significant prognostic value. Notably, in both MTV and TLG analyses, two studies contributed a weight of 0%, reflecting limited precision and highlighting the need for larger data. **Conclusions:** Baseline [^18^F]FDG-PET parameters such as SUVmax and SUVmean showed a potential prognostic value in patients with TETs. However, these results are based on a limited number of retrospective studies with significant heterogeneity. Prospective multicentre investigations are necessary to confirm the potential role of [^18^F]FDG-PET for risk stratification in TETs.

## 1. Introduction

Thymic epithelial tumours (TETs) are a heterogeneous group of thoracic malignancies originating from the thymic epithelium with distinct biological behaviours and prognoses [1]. Although rare, they represent the most common tumours of the anterior mediastinum in adults (approximately 50% of all anterior mediastinal masses) [1,2,3]. The annual incidence is approximately 1 in 769,000, and the male-to-female ratio is 1:1.4. Usually, the age of onset is between 30 and 70 (with a mean age of 50), but in some rare cases they can also appear during childhood [4]. While half of patients can be asymptomatic, others can experience chest symptoms, such as dyspnoea, chest pain, upper respiratory tract infections, fatigue, weight loss, prominent neck veins, and cough or pneumonia. Thymomas are often associated (in 20–40% of patients) with myasthenia gravis, an autoimmune disease that manifests as double vision, ptosis, dysphagia, and weakness [5,6]. Based on histology, thymomas are primarily classified as low-risk thymomas (A, AB and B1), high-risk thymomas (B2, B3), and thymic carcinomas (TC) [1]. In addition, functional thymic neuroendocrine tumours have been also described [7,8,9].

Surgery plays a primary role in the management of patients in whom complete resection is feasible. The benefit of post-operative radiotherapy (PORT) is still controversial, since it could be related to stage, histotype, and preoperative chemotherapy. If the tumour is unresectable at diagnosis, radiotherapy or concurrent chemoradiotherapy is the most used approach. More recently, immune checkpoint inhibitor therapy has shown promising results in TETs patients [10,11,12,13,14].

For these reasons, accurate lesion identification and staging is crucial for treatment planning and prognostication. Currently the Masaoka–Koga and TNM systems are commonly used for this purpose [1,15,16].

In this context, multimodal imaging plays a pivotal role. Usually, after chest radiography, Computed Tomography (CT) is the most common modality used to characterize mediastinal masses and for proper patient staging. However, in some cases, Magnetic Resonance Imaging (MRI) provides high soft tissue contrast and tissue characterization, allowing further evaluation of indeterminate mediastinal lesions. Specifically compared to CT, MRI can better differentiate cystic from solid lesions, as well as thymic hyperplasia from thymic malignancy, thanks to its own ability to identify and detect the fat component [17,18,19,20].

The role of [^18^F]Fluorodeoxyglucose positron emission tomography ([^18^F]FDG-PET) in TETs is debated, despite its increased use.

[^18^F]FDG-PET is not generally recommended in the definition of thymic masses [21], due to its intrinsic limited value in the differential diagnosis from other mediastinal entities, such as primary mediastinal lymphomas [22,23,24]. In addition, increased thymic [^18^F]FDG uptake could be physiologically detected especially in young people, or alternatively could indicate the presence of thymic hyperplasia, also as a rebound of external stress, like in patients under chemotherapy [25,26]. However, [^18^F]FDG-PET can provide valuable diagnostic information and it is frequently used to complete the staging work-up, especially in case of tumours with aggressive histology and an advanced stage [21].

Currently, the added value of [^18^F]FDG-PET lies to the possibility to extract some semi-quantitative data that could potentially play a role as a prognostic marker, as well as help in the evaluation of primary mediastinal masses.

Maximum standardized uptake value (SUVmax), the most used and accessible PET parameter in clinical practice, has already been reported by multiple studies as a potential predictive biomarker for differentiating low-risk thymoma from high-risk thymoma and TC [27,28,29,30,31]. However, above the most quoted SUVmax, other semiquantitative parameters, such as mean SUV (SUVmean), Metabolic Tumour Volume (MTV), and Total Lesion Glycolysis (TLG) are gaining increasing interest in the scientific community to noninvasively differentiate benign tumours from malignant mediastinal ones [32]. In addition, the progressive and rapid evolution of imaging acquisition, processing and analyses, could also open the way for a better understanding of tumour behaviour. In this scenario, machine learning and texture analysis, which comprises a variety of mathematical methods for calculating indices that describe the relationships between grey-level pixel intensity and their spatial distribution within an image, have been increasingly applied in clinical studies. In the setting of TETs, preliminary results showed the potential ability of [^18^F]FDG-PET to differentiate between different tumour grades, by combining SUVmax and with measures of intratumoural heterogeneity, thus possibly allowing for a more tailored “non-invasive” diagnosis, risk stratification, and staging of these rare tumours [33,34,35].

Despite the increasing clinical use of [^18^F]FDG-PET, its prognostic value in TETs remains scarcely explored. In fact, the current literature is mainly focused on the diagnostic role of PET or on the histological stratification of TETs, without quantitatively addressing progression-free survival (PFS). Therefore, the present systematic review and meta-analysis specifically aimed to evaluate the prognostic role of baseline [^18^F]FDG-PET-derived parameters in predicting PFS in patients with TETs, providing a synthesis of the available evidence and identifying current limitations.

## 2. Materials and Methods

### 2.1. Research Strategy

The present study was conducted in accordance with the Preferred Reporting Items for Systematic Reviews and Meta-Analyses (PRISMA) statement [36]. No pre-defined protocol was registered. The literature search was performed in PubMed, Embase, and Scopus databases from inception to January 2025, with an update conducted in May 2025. Adopted research strings are reported in Table 1.

### 2.2. Study Selection

Two independent reviewers (AM and FL) conducted the screening and selection of studies using the Rayyan platform [37]. Discrepancies were resolved with consensus. We included only studies that evaluated the prognostic value of baseline [^18^F]FDG-PET-derived semiquantitative parameters in patients with TETs. The outcome considered was PFS, encompassing either disease progression, or recurrence or death as events. In the case of more than one publication with an overlapping patients’ sample, we only considered the most recent work. Only studies providing hazard ratios (HRs) and relative 95% confidence intervals (95% CIs) were included. Sub-group or post-hoc analyses from other studies, case reports and abstract only were excluded. Only manuscripts written in English were considered.

### 2.3. Data Extraction

For each study, data were independently collected by two reviewers (AM and FL), and discrepancies were solved by consensus. The extracted information included author, year, study design, number of enrolled patients included in survival analyses, follow-up duration, TETs WHO classification, TETs stage, [^18^F]FDG-PET-derived semiquantitative parameters and their computing modalities, and the events during follow-up (for PFS calculation). For each [^18^F]FDG-PET parameter investigated as a predictor of PFS in at least two different studies, HRs and respective 95% CIs from univariate and (if performed or available) multivariate analyses were also reported. When imaging variables were tested as protective factors (i.e., lower values associated with better outcomes), HRs and CIs were transformed so that higher values of metabolic parameters would reflect a negative association with PFS.

In studies applying more than one method for image analyses, the PET-derived measures obtained with the more extensively validated approach were collected.

### 2.4. Statistical Analysis

Forest plots were generated to visualize pooled HRs for the association between PET-based measures and PFS. Statistical heterogeneity between studies included in the meta-analysis was assessed with the Cochrane’s Q statistic [38]. The I^2^ statistics were used to describe the proportion of interstudy variation caused by heterogeneity [39]. When substantial heterogeneity was detected (*p* < 0.1), the pooled estimates were calculated with the DerSimonian random-effect model, while the fixed-effect model was adopted in cases of no substantial heterogeneity. When applicable, sub-group analyses were performed and sensitivity analyses were adopted using the leave-one-out approach [40]. The assessment of non-reporting bias and Egger test were not performed as no analysis included more than ten studies [41]. The RevMan software version 5.3 was used for statistical analyses [42]. Statistical significance was set at *p* < 0.05 (two-tailed).

### 2.5. Risk of Bias (RoB) Assessment

The quality of the included studies was independently evaluated by two reviewers (AM and FL) with the Newcastle–Ottawa Scale [43]. The two authors resolved any discrepancies by consensus. The robvis tool was used to create RoB graphs [44].

## 3. Results

**Figure 1:**
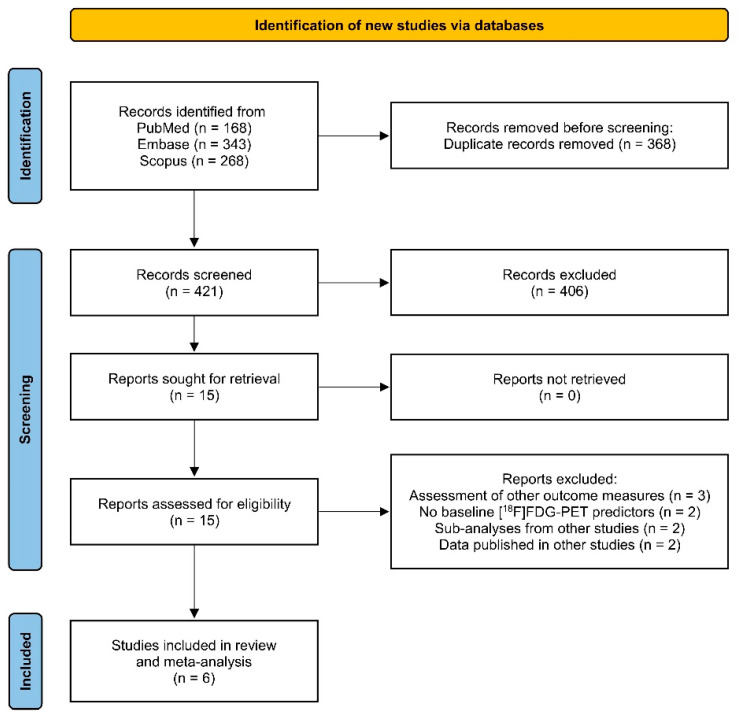
PRISMA flow-diagram.

As displayed in the PRISMA flow-diagram (Figure 1), 421 records were screened. After removal of duplicate records, screening, and full-text assessment, six manuscripts were considered eligible for systematic review and meta-analysis [45,46,47,48,49,50].

For included studies, precise design, number of patients, follow-up duration, TETs classification, and clinical outcomes defined as events for PFS are summarized in Table 2. The cumulative number of patients was 593. All studies had a retrospective design, with the number of patients ranging from 42 to 177. One single study collected patients form two centres, while all the others were monocentric.

All [^18^F]FDG-PET semiquantitative measures tested as predictors for PFS are displayed in Table 3, along with the availability of HRs (95% CI) from univariate or multivariate analyses. All studies assessed the prognostic power of the SUVmax, while its SUVmean was evaluated in half of the included works [45,49,50]. MTV and TLG were tested as predictors of PFS in five studies [45,46,47,49,50], but in one of them HRs for MTV and TLG were not reported [50]. For the computation of MTV, and subsequently of TLG, as the product of MTV and SUVmean, a fixed SUV threshold of 2.5 was adopted in three studies [45,47,49], while 40% of the SUVmax was used as threshold in the other two works [48,50]. PET-based findings reported only by a single study were not included in the analysis. PET measures with respective HRs and CIs are shown in Supplementary Table S1.

### 3.1. Narrative Description

In 2021 Lee et al. [45] conducted a retrospective study that included 83 patients (21 low-risk thymomas (25.3%), 27 high-risk thymomas (32.5%), and 35 thymic carcinomas (42.2%)) to investigate the prognostic value of volume-based [^18^F]FDG-PET and clinical parameters. Recurrence or disease progression occurred in 24 patients (28.9%). On univariate analysis, higher Masaoka stage (*p* < 0.001), aggressive histological type (*p* = 0.009), treatment modality (Surgery and/or adjuvant therapy vs. non-surgical; *p* = 0.001), and higher SUVmax, SUVmean, MTV, and TLG (all *p* < 0.001) were significantly associated with shorter PFS. In addition, only SUVmean (*p* < 0.001) and Masaoka stage (*p* = 0.001) resulted as independent prognostic factors for PFS on multivariate analysis.

In the same year, Li et al. [46] investigated clinical and [^18^F]FDG-PET metabolic parameters in 42 treatment-naive patients with TETs. Data on tumour-infiltrating cells were also collected and analysed. Higher SUVmax, MTV, and TLG were observed in advanced Masaoka–Koga stages compared to early-stage disease, and higher SUVmax was noted in advanced-TNM-stage disease compared to early stage. Interestingly, higher SUVmax was extracted from lesions with lower CD4-positive tumour-infiltrating lymphocytes.

Han et al. [47] retrospectively enrolled a total of 186 consecutive patients with resectable TETs who underwent preoperative [^18^F]FDG-PET (145 thymomas, 41 thymic carcinomas). Fully Automatic Quantitative Measurement using a Convolutional Neural Network was utilized to analyse the [^18^F]FDG-PET. Automatically measured SUVmax, MTV, and TLG were in good agreement with manual measurements and showed good diagnostic accuracy for thymic carcinoma (AUCs: SUVmax, 0.95; MTV, 0.85; TLG, 0.87) and significant prognostic value (HRs: SUVmax, 1.31 [95% CI, 1.16–1.48]; MTV, 2.11 [95% CI, 1.09–4.06]; TLG, 1.90 [95%CI, 1.12–3.23]).

In 2024, the group of Akamine [48] retrospectively evaluated 177 patients with resectable TETs who preoperatively underwent [^18^F]FDG-PET. Among them, 145 (81.9%) had pathological early-stage TET (stage I or II) while 32 (19.1%) had advanced stage (stage III or IV). For the advanced stage group, lymph node (LN) metastases were preoperatively detected by [^18^F]FDG-PET in 30.8% of patients with a SUVmax > 5.9 of the primary tumour with subsequent confirmed pathological LN positivity, whereas LN metastases were not pathologically detected in patients with a SUVmax < 5.9 of the primary. Moreover, SUVmax > 5.6 was associated with a general worse prognosis for PFS. In addition, in patients with advanced-stage TETs, LN recurrence was significantly more frequent among those classified as N1 at preoperative [18F]FDG-PET (75.0% vs. 7.1%).

Chao et al. [49] evaluated the impact of intratumoural metabolic heterogeneity and quantitative [^18^F]FDG-PET imaging parameters in predicting patient outcomes in 100 patients with TETs. The univariate analysis showed that Masaoka stage, TNM stage, WHO classification, SUVmax, SUVmean, TLG, and a heterogeneity index (HI: standard deviation [SD] divided by SUVmean) were significant prognostic factors for PFS). Subsequently, multivariate analyses confirmed that HI (*p* < 0.001) and TNM stage (*p* = 0.002) were independent prognostic factors for PFS. The authors also, demonstrated that TNM stage, WHO classification, SUVmax, and HI were significant prognostic factors for overall survival (OS) in the univariate analysis, while TNM stage remained an independent prognostic factor for OS even in the multivariate analysis (*p* = 0.024). Specifically, the Kaplan Meier analyses showed worse prognoses for patients with TNM stages III and IV and HI ≥ 0.16 compared to those with stages I and II and HI < 0.16 (log-rank *p* < 0.001).

Recently, Pizzuto et al. [50] retrospectively analysed 116 patients (49/67 M/F; mean age 59.5 years) who underwent preoperative [^18^F]FDG-PET followed by thymectomy. In total, 27 thymic hyperplasia, 41 low-risk thymomas (types A, AB, and B1), and 48 high-risk thymomas (B2, B3 thymoma, and carcinoma) were included. SUVmax, SUVmean, SUVpeak, as well as the ratio between SUVmax of the target lesion and the liver SUVmax (rPET), quotient of SUVpeak in the tumour residual (qPET), and tumour to mediastinum ratio (T/M) were significantly higher in high-risk thymomas than low-risk and hyperplasia (*p* < 0.001). TLG and MTV were significantly higher in patients with low-risk thymomas (*p* < 0.001). Moreover, SUVmax, SUVmean, and SUVpeak cutoffs of <4.3, <2.87, and 4.03, respectively, significantly distinguished patients with longer time to recurrence (*p* = 0.009, *p* = 0.05, and *p* = 0.05, respectively).

### 3.2. Prognostic Value of SUVmax

**Figure 2:**
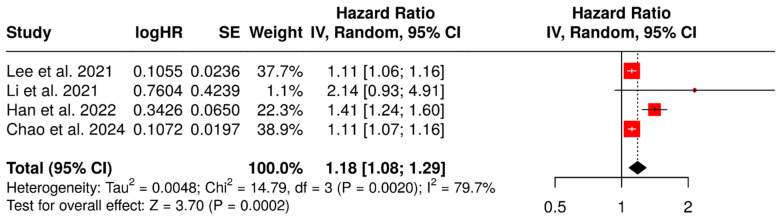
SUVmax (continuous variable) as a predictor of PFS [45,46,47,49].

When considered as a continuous variable, SUVmax resulted in a significant predictor of PFS (random-effect HR: 1.18, 95% CI: 1.08–1.29, *p* < 0.001) (Figure 2). Significant heterogeneity was detected (χ^2^ = 14.79, *p* = 0.002; I^2^ = 79.7%). This result was confirmed by the leave-one-out sensitivity analyses (Supplementary Figure S1), even when only considering the two studies with a low risk of bias (Supplementary Figure S2).

**Figure 3:**
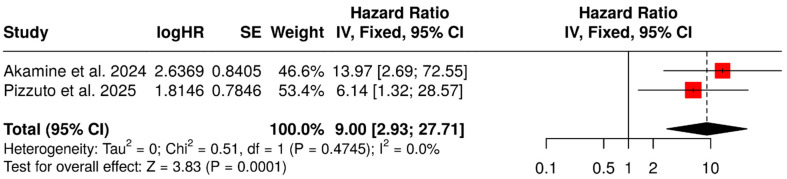
SUVmax (dichotomous variable) as a predictor of PFS [48,50].

The analysis of the two studies assessing SUVmax as a dichotomous variable showed that the group of patients with higher values was associated with worse PFS (fixed-effect HR: 9.00, 95% CI: 2.93–27.71, *p* < 0.001) (Figure 3). No significant heterogeneity was detected (χ^2^ = 0.51, *p* = 0.475; I^2^ = 0.0%).

### 3.3. Prognostic Value of SUVmean

**Figure 4:**
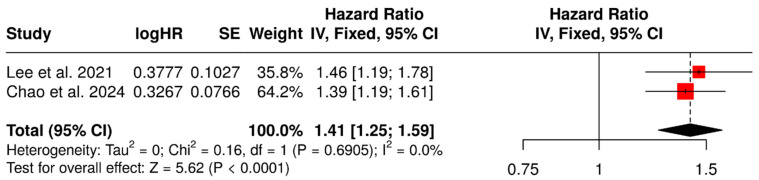
SUVmean (continuous variable) as a predictor of PFS [45,49].

The two studies evaluating SUVmean as a continuous predictor showed a significant association between this metabolic parameter and a shorter PFS (fixed-effect HR: 1.41,95% CI: 1.25–1.59, *p* < 0.001) (Figure 4). No significant heterogeneity was detected (χ^2^ = 0.16, *p* 0.691; I^2^ = 0.0%).

### 3.4. Prognostic Value of MTV and TLG

**Figure 5:**
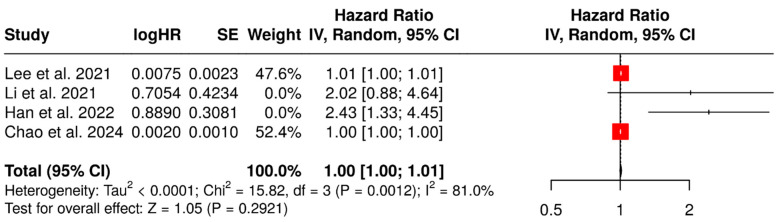
MTV (continuous variable) as a predictor of PFS [45,46,47,49].

**Figure 6:**
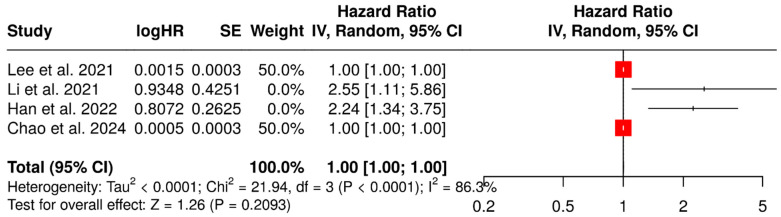
TLG (continuous variable) as a predictor of PFS [45,46,47,49].

Four studies evaluated both MTV and TLG as continuous predictors of PFS. These two [^18^F]FDG-PET-based parameters showed no significant association with PFS (*p* = 0.292 for MTV; *p* = 0.209 for TLG) (Figure 5 and Figure 6, respectively). No significant association was detected even considering only studies with a fixed SUV threshold for MTV contouring (Supplementary Figure S3).

## 4. Quality of the Studies

**Figure 7:**
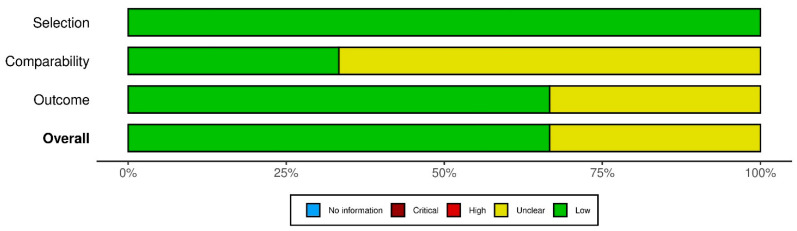
Pooled risk of bias of included studies.

Pooled RoB data are displayed in Figure 7. The overall RoB was unclear in two studies, due to weaknesses in the Comparability and Outcome domains, while the remaining four publications showed an overall low RoB. As shown in Supplementary Figure S4, four studies had an unclear risk of bias in the Comparability domain, and two studies were unclear in the Outcome domain.

## 5. Discussion

Among baseline [^18^F]FDG-PET semiquantitative parameters in patients with TETs, the present systematic review and meta-analysis identified SUVmax and SUVmean as significant predictors of PFS, while MTV and TLG did not demonstrate a statistically significant prognostic value. The association between higher SUVmax and worse PFS was consistent across most studies, both when treated as a continuous and dichotomous variable.

These findings are in line with previous literature on other cancers [51,52,53,54,55], underscoring this metabolic parameter as a potentially simple and widely available prognostic biomarker in clinical practice. In fact, SUVmax reflects areas of highest metabolic activity, often correlating with increased cellular proliferation and hypoxia, which are known drivers of treatment resistance and poorer outcomes. However, it should be noted that only two studies assessing SUVmax as a dichotomous variable were included [48,50], thus leading to uncertainty in the results of this analysis.

Thymic carcinoma showed higher SUVmax than high-grade thymomas, which in turn presented higher [^18^F]FDG uptake than low-grade thymomas [29,30]. Among the included studies, one evaluated only patients with thymic carcinoma [46], another two [47,48] presented HRs for SUVmax at multivariate analysis, and the remaining three showed univariate HRs for SUVmax [45,49,50]. Thus, it is not possible to define SUVmax as a prognostic factor independent from other characteristics influencing the clinical outcome in these patients.

SUVmean was identified as an additional metabolic parameter significantly associated with poorer PFS, suggesting that overall [^18^F]FDG uptake, rather than only the maximum, could hold further prognostic value. Despite few previous findings highlighting the prognostic role of SUVmean in other clinical settings [56,57], in our work only two studies were assessable, and more data are necessary to confirm this finding.

From a clinical standpoint, these results suggest that patients with high baseline SUV values may require intensified treatment strategies as well as tailored follow-up programs with closer surveillance intervals. Potentially, integrating metabolic information with morphologic staging systems could help refine risk stratification and guide clinical decision-making in this rare disease setting.

Conversely, neither MTV nor TLG demonstrated significant associations with PFS. This lack of prognostic relevance disagrees with a wide literature about several solid tumours [58,59,60,61,62], and it might likely be due to some confounding factors. Firstly, these volumetric parameters are more susceptible to inter-study variability in term of segmentation methods and thresholds, which may reduce reproducibility and consistency across studies. In particular, three of the four considered studies used a fixed SUV threshold for volumetric segmentation [45,47,49], while the fourth used a variable threshold set at 40% of SUVmax [46]. Moreover, in both analyses for MTV and TLG, two studies contributed a weight of 0% to the pooled estimates, highlighting the necessity of a larger amount of evidence on this topic to obtain reliable results. Further considerations are that TETs are confined in a rigid structure with little expansion, such as the mediastinum, and that even low-risk thymoma can have a huge volume. Tumour heterogeneity, rather than volumetric parameters, could therefore correlate better with PFS. Imaged tumour heterogeneity is thought to result from regional differences in tumour cellularity, proliferation, hypoxia, angiogenesis, and necrosis, all of which are related to tumour grade [63,64]. At the same volume, aggressive tumours are often associated with intratumoural hypoxia or necrosis [65], both of which can manifest as heterogeneous tumour uptake on [^18^F]FDG PET, with also areas of low [^18^F]FDG uptake.

This study has several limitations. Firstly, no protocol was registered, which may undermine the reproducibility of this meta-analysis. Secondly, the limited number of studies did not allow subgroup analyses according to tumour type or overall risk of bias in all cases. Additionally, the small number of studies prevented a robust assessment of non-reporting bias, and the presence of publication bias cannot be excluded. Furthermore, the substantial heterogeneity observed between studies may limit the reliability of our findings. Moreover, all included studies had a retrospective design, and all except one were monocentric. Prospective, multicentric studies are necessary to clarify the prognostic role of [^18^F]FDG-PET in patients with TETs. The prognostic performance of PET measures was assessed across different histopathological variants, and in most cases multivariable analyses were not performed, leaving the independence of metabolic parameters as predictors of PFS unclear. Finally, baseline [^18^F]FDG-PET is not always performed in patients with TETs, limiting the generalizability of our findings across all clinical scenarios.

Generally, there are still several challenges in using [^18^F]FDG-PET semiquantitative parameter assessment for routine clinical practice that need to be addressed, such as variations between instruments and institutions, leading to lower reproducibility. Harmonization efforts should be implemented to promote greater uniformity in clinical practice. The limited literature evidence about the prognostic role of [^18^F]FDG-PET in TETs highlights the need of larger multi-institutional studies to confirm our preliminary findings, especially considering the rarity of these diseases [66].

Looking ahead, the progressive integration of radiomics and artificial intelligence methods may play a pivotal role in overcoming current limitations. Radiomic approaches allow for the extraction of high-dimensional quantitative features beyond conventional PET parameters, potentially uncovering novel imaging biomarkers with prognostic value. When combined with AI-based algorithms, these data could support risk stratification and personalized treatment strategies. Future multicentre, prospective validation studies should therefore incorporate radiomics and AI-driven analyses to enhance reproducibility, improve prognostic accuracy, and ultimately foster their translation into clinical practice.

## 6. Conclusions

Our findings support baseline [^18^F]FDG-PET-derived measures, such as SUVmax and SUVmean, as possible predictors of PFS in patients with TETs, suggesting their role as non-invasive biomarkers for risk stratification, identification of personalized follow-up strategies or more aggressive therapeutic approaches in this setting.

Conversely, the study results challenged the prognostic value of volumetric parameters, such as MTV and TLG, on PFS. Nevertheless, all available studies are limited by a retrospective design and single or bi-centric settings. Further prospective studies in larger multicentric samples are necessary to clearly establish the prognostic impact of [^18^F]FDG-PET in these patients.

## Figures and Tables

**Table 1:** Literature search strategy.

Database	Research String
**PubMed**	((thymoma) OR ((thymic) AND (cancer OR carcinoma OR adenocarcinoma OR neoplasia OR neoplasm OR tumour OR tumour)) AND ((positron emission tomography OR pet) AND (fdg OR fluorodeoxyglucose)) AND (survival OR progression OR recurrence OR relapse OR prognosis OR prognostic))
**Embase**	((‘thymoma’) OR ((‘thymic’) AND (‘cancer’ OR ‘carcinoma’ OR ‘adenocarcinoma’ OR ‘neoplasia’ OR ‘neoplasm’ OR ‘tumour’ OR ‘tumour’)) AND ((‘positron emission tomography’ OR ‘pet’) AND (‘fdg’ OR ‘fluorodeoxyglucose’)) AND (‘survival’ OR ‘progression’ OR ‘recurrence’ OR ‘relapse’ OR ‘prognosis’ OR ‘prognostic’))
**Scopus**	((thymoma) OR ((thymic) AND (cancer OR carcinoma OR adenocarcinoma OR neoplasia OR neoplasm OR tumour OR tumour)) AND ((“positron emission tomography” OR pet) AND (fdg OR fluorodeoxyglucose)) AND (survival OR progression OR recurrence OR relapse OR prognosis OR prognostic))

**Table 2:** Characteristics of the included studies.

Author, Year	Study Design	Enrolled Patients	Follow-Up (Months)	TETs Classification	Event for PFS
Lee et al. 2021 [45]	Retrospective, monocentric	83	Mean: 28.6 SD: 22.2 Range: 0.0–79.0	Thymoma Carcinoma	Disease recurrence, progression, or death
Li et al. 2021 [46]	Retrospective, monocentric	42	Mean: 21 SD: 12 Range: 7–60	Carcinoma	Disease progression
Han et al., 2022 [47]	Retrospective, monocentric	114	Median: 39 IQR: 25–58	Thymoma Carcinoma	Disease recurrence
Akamine et al., 2024 [48]	Retrospective, monocentric	177	Median: 35 IQR: 12–59	Thymoma Carcinoma Neuroendocrine neoplasm	Disease recurrence, progression, or death
Chao et al., 2024 [49]	Retrospective, monocentric	100	Mean: 25.7 SD: 19.8 Range 1–97	Thymoma Carcinoma	Disease recurrence, progression, or death
Pizzuto et al., 2025 [50]	Retrospective, bi-centric	77	Median: 38 Range 14–72	Hyperplasia Thymoma Carcinoma	Disease recurrence

**Table 3:** [^18^F]FDG-PET parameters reported in the included studies.

Author, Year	[^18^F]FDG-PET Parameter	Variable Type	Variable Computation
Lee et al., 2021 [45]	SUVmax	Continuous	
	SUVmean	Continuous	
	MTV	Continuous	Fixed SUV threshold of 2.5
	TLG	Continuous	
Li et al., 2021 [46]	SUVmax	Continuous	
	Primary tumour MTV	Continuous	Threshold at 40% of SUVmax
	Primary tumour TLG	Continuous	
	Metastases MTV	Continuous	Threshold at 40% of SUVmax
	Metastases TLG	Continuous	
Han et al., 2022 [47]	SUVmax	Continuous	
	MTV	Continuous	Fixed SUV threshold of 2.5
	TLG	Continuous	
	SUVmax	Continuous	Automatic segmentation
	MTV	Continuous	Automatic segmentation Fixed SUV threshold of 2.5
	TLG	Continuous	Automatic segmentation
Akamine et al., 2024 [48]	SUVmax	Binarized	
Chao et al., 2024 [49]	SUVmax	Continuous	
	SUVmean	Continuous	
	MTV	Continuous	Fixed SUV threshold of 2.5
	TLG	Continuous	
	Heterogeneity index-1	Continuous	
	Heterogeneity index-2	Continuous	
Pizzuto et al., 2025 [50]	SUVmax	Binarized	
	SUVmean	Binarized	
	SUVpeak	Binarized	
	MTV	Binarized	Threshold at 40% of SUVmax
	TLG	Binarized	
	rPET	Binarized	
	qPET	Binarized	
	T/M	Binarized	

Legend: Standardized uptake value (SUV), metabolic tumour volume (MTV), total lesion glycolis (TLG), Positron emission tomography (PET).

## Data Availability

The original contributions presented in this study are included in the article/Supplementary Materials. Further inquiries can be directed to the corresponding author.

## Supplementary Materials

The following supporting information can be downloaded at: https://www.mdpi.com/article/10.3390/diagnostics15192458/s1, PRISMA Checklist. Table S1. Hazard ratios (HRs) and 95% confidence intervals (CIs) of [^18^F]FDG-PET measures as predictors of the progression-free survival (PFS) at uni- or multivariate Cox regression analyses extracted from the included studies [45,46,47,48,49,50]. Supplementary Figure S1. SUVmax (continuous variable) as a predictor of the progression-free survival (PFS) with the leave-one-out approach as a sensitivity analysis [45,46,47,49]. Figure S2. SUVmax (continuous variable) as a predictor of the progression-free survival (PFS) including only studies with low risk of bias as a sensitivity analysis [46,47]. Figure S3. MTV (continuous variable) as a predictor of the progression-free survival (PFS) including only studies with a fixed SUV threshold for contouring [45,47,49]. Figure S4. Newcastle-Ottawa Scale for risk of bias (RoB) assessment of included studies [45,46,47,48,49,50].
