# Supplementary material for: The Value of Baseline [18F]FDG-PET in Predicting the Progression-Free Survival in Patients with Thymic Epithelial Tumours: A Systematic Review and Meta-Analysis
Source: Diagnostics (Basel). 2025 Sep 26;15(19):2458. doi: 10.3390/diagnostics15192458 (PMC12523372; doi:10.3390/diagnostics15192458)

Alberto Miceli [1,*], Maria Librando [2], Francesco Dondi [3], Lorenzo Jonghi-Lavarini [4], Adriana D'Antonio [5], Antonio Mura [6], Anna Giulia Nappi [7], Guido Rovera [8], Maria Silvia De Feo [9], Giulia Santo [10] and Francesco Lanfranchi [11]

[1] Nuclear Medicine Unit, Azienda Ospedaliero-Universitaria SS. Antonio e Biagio e Cesare Arrigo, 15121 Alessandria, Italy
[2] Nuclear Medicine Unit, Department of Biomedical and Dental Sciences and Morpho-Functional Imaging, University of Messina, 98122 Messina, Italy; marialibrando@hotmail.it
[3] Division of Nuclear Medicine, Università degli Studi di Brescia and ASST Spedali Civili di Brescia, 25123 Brescia, Italy; francesco.dondi@unibs.it
[4] Department of Radiotherapy and Nuclear Medicine, ASST-Cremona, 26100 Cremona, Italy; lorenzomaria.jonghi-lavarini@asst-cremona.it
[5] Department of Advanced Biomedical Sciences, University Federico II, 80131 Naples, Italy; a.dantonio62@gmail.com
[6] Unit of Nuclear Medicine, Department of Medicine, Surgery and Pharmacy, University of Sassari, 07100 Sassari, Italy; a.mura203@studenti.uniss.it
[7] Nuclear Medicine Unit, Interdisciplinary Department of Medicine, University of Bari "Aldo Moro", Piazza Giulio Cesare 11, 70124 Bari, Italy; anna.giulia.nappi@gmail.com
[8] Department of Medical Sciences, University of Turin, 10126 Turin, Italy; guido.rovera@unito.it
[9] Department of Radiological Sciences, Oncology and Anatomo Pathology, Sapienza University of Rome, 00151 Rome, Italy; mariasilvia.defeo@uniroma1.it
[10] Department of Experimental and Clinical Medicine, "Magna Graecia" University of Catanzaro, 88100 Catanzaro, Italy; giulia.santo@unicz.it
[11] Department of Experimental Medicine (DIMES), University of Genoa, 16132 Genoa, Italy; dr.francescolanfranchi@gmail.com
* Correspondence: alberto.miceli@ospedale.al.it

Supplementary Table S1. Hazard ratios (HRs) and 95% confidence intervals (CIs) of [^{18}F]FDG-PET measures as predictors of the progression-free survival (PFS) at uni- or multivariate Cox regression analyses extracted from the included studies [45, 46, 47, 48, 49, 50].

		Univariate analysis			Multivariate analysis		
			95% CI			95% CI	
**Author, year**	**[^{18}F]FDG-PET measure**	**HR**	**Lower**	**Upper**	**HR**	**Lower**	**Upper**
Lee et al. 2021	SUVmax (continuous)	1.111	1.061	1.164			
Li et al. 2021	SUVmax (continuous)	2.139	0.932	4.910			
Han et al. 2022	SUVmax (continuous)	1.39	1.24	1.57	1.40	1.24	1.60
Akamine et al. 2024	SUVmax (binarized)	28.24	6.48	123.07	13.96	2.69	72.55
Chao et al. 2024	SUVmax (continuous)	1.113	1.071	1.157			
Pizzuto et al. 2025	SUVmax	6.135	1.319	28.571			
Lee et al. 2021	SUVmean (continuous)	1.403	1.195	1.647	1.459	1.193	1.784
Chao et al.	SUVmean (continuous)	1.386	1.193	1.611			
Pizzuto et al., 2025	SUVmean (binarized)	3.636	0.962	13.699			
Lee et al. 2021	MTV (continuous)	1.007	1.003	1.012			
Li et al. 2021	MTV (continuous)	2.025	0.883	4.642			
Han et al. 2022	MTV (continuous)	2.430	1.330	4.450			
Chao et al. 2024	MTV (continuous)	1.002	1.000	1.004			
Lee et al. 2021	TLG (continuous)	1.002	1.001	1.002			
Li et al. 2021	TLG (continuous)	2.546	1.107	5.859			
Han et al. 2022	TLG (continuous)	2.240	1.340	3.750			
Chao et al. 2024	TLG (continuous)	1.000	1.000	1.001			

Supplementary Figure S1. SUVmax (continuous variable) as a predictor of the progression-free survival (PFS) with the leave-one-out approach as a sensitivity analysis [45, 46, 47, 49].

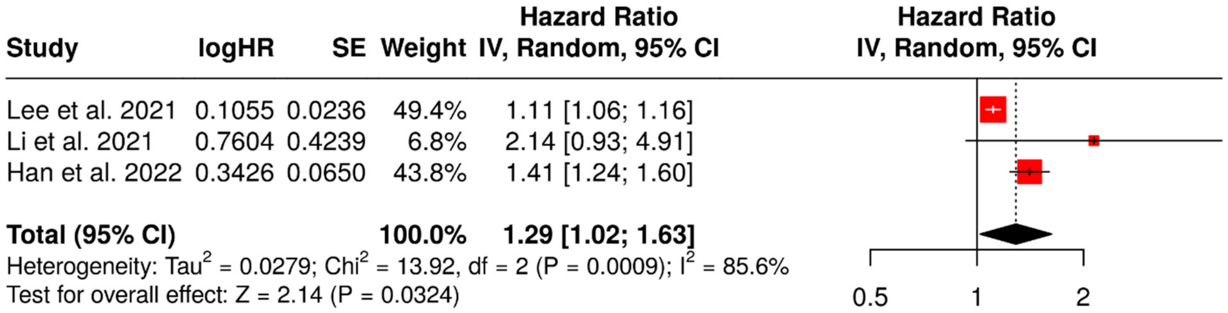

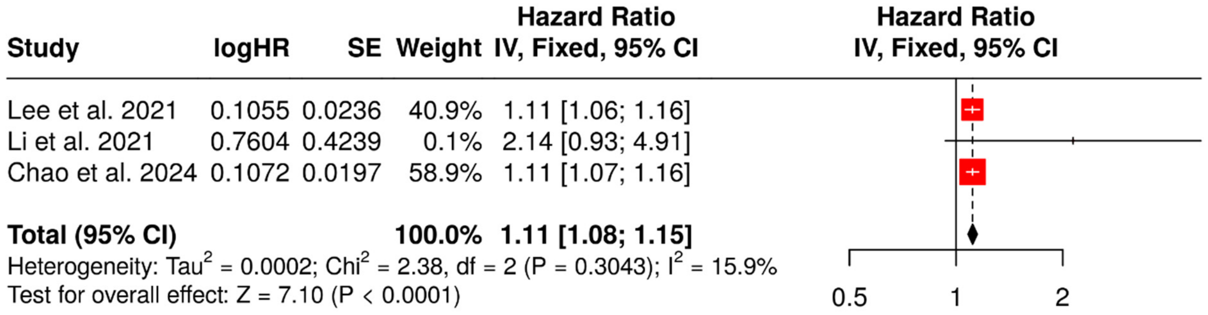

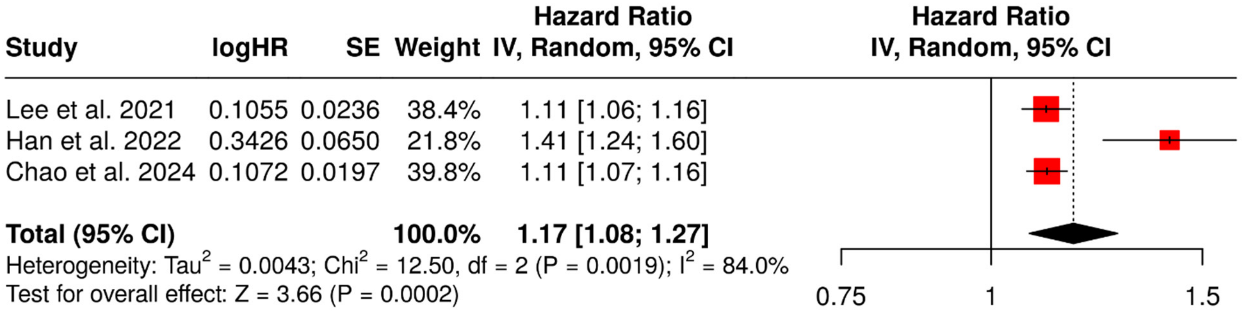

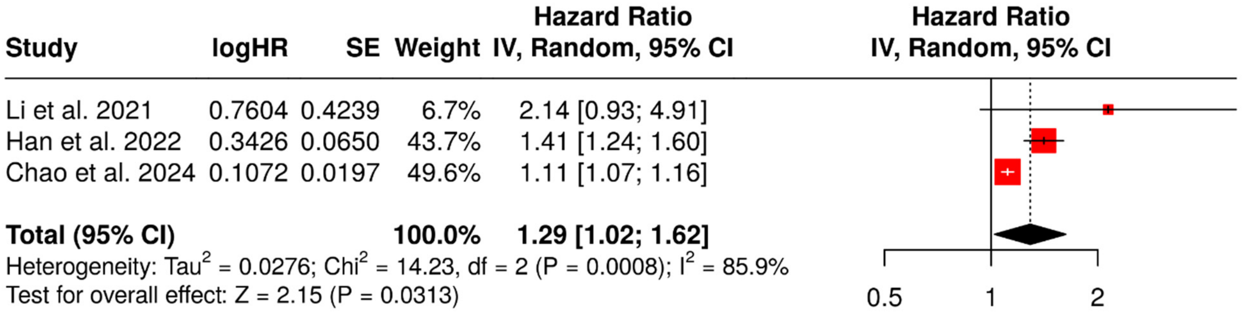

Supplementary Figure S2. SUVmax (continuous variable) as a predictor of the progression-free survival (PFS) including only studies with low risk of bias as a sensitivity analysis [46, 47].

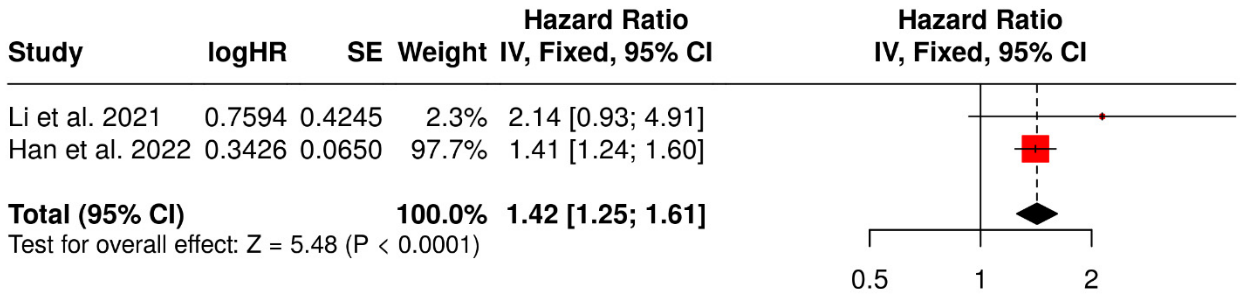

Supplementary Figure S3. MTV (continuous variable) as a predictor of the progression-free survival (PFS) including only studies with a fixed SUV threshold for contouring [45, 47, 49].

Study	logHR	SE	Weight	Hazard Ratio IV, Random, 95% CI
Lee et al. 2021	0.0075	0.0023	47.2%	1.01 [1.00; 1.01]
Han et al. 2022	0.8890	0.3081	0.0%	2.43 [1.33; 4.45]
Chao et al. 2024	0.0020	0.0010	52.8%	1.00 [1.00; 1.00]
**Total (95% CI)**			**100.0%**	**1.00 [1.00; 1.01]**

Heterogeneity: $Tau^2 < 0.0001$; $Chi^2 = 13.07$, df = 2 (P = 0.0015); $I^2 = 84.7\%$

Test for overall effect: Z = 1.10 (P = 0.2722)

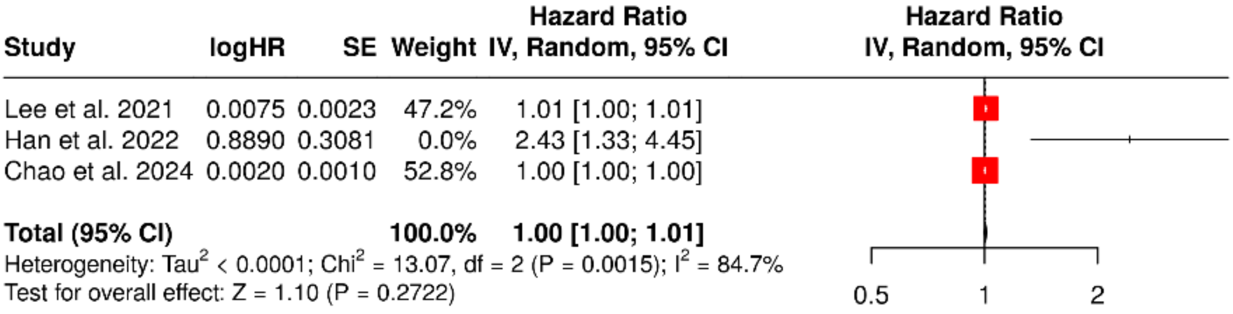

Supplementary Figure S4. Newcastle-Ottawa Scale for risk of bias (RoB) assessment of included studies [45,46, 47, 48, 49, 50].

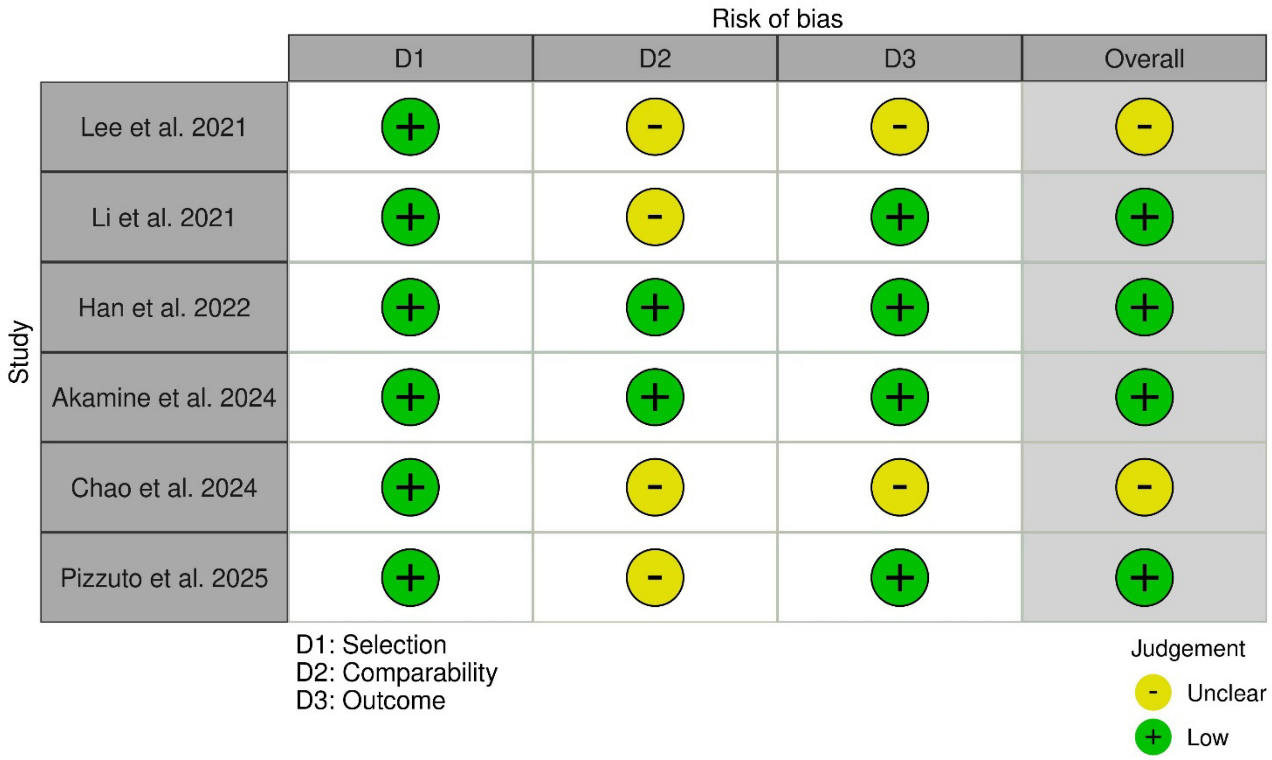

Supplement: Supplementary file 1 [file diagnostics-15-02458-s001.zip › diagnostics-3809717-supplementary.pdf]
